# Theater testing a sexual and reproductive health program for Latina teens and their female caregivers: a mixed methods study

**DOI:** 10.1186/s12889-025-22233-1

**Published:** 2025-07-07

**Authors:** Katherine G. Merrill, Jacqueline Silva, Jaime Atadero, Ivy Lee Hung, Susana Salgado, Jennifer K. Cano, Veronica Nabor, Angela Sedeño, Sara Vargas, Gisel Romero, Carol Perez, Jamison Merrill, Jeff DeCelles, Jacqueline Fuentes, Kate Florence, Laura Rodriguez, Corin Mora, Ana A. Baumann, Kate Guastaferro, Geri R. Donenberg

**Affiliations:** 1 Center for Dissemination and Implementation Science, University of Illinois Chicago, Chicago, IL USA; 2 Floreciendo Community Advisory Council, Chicago, IL USA; 3 Centro Romero, Chicago, IL USA; 4 The Kedzie Center, Chicago, IL USA; 5 LoSAH Center of Hope, Chicago, IL USA; 6 School of Public Health, University of Illinois Chicago, Chicago, IL USA; 7 Waves for Change, Cape Town, South Africa; 8 Division of Public Health Sciences, Department of Surgery, Washington University in St. Louis, St. Louis, MO USA; 9 School of Global Public Health, New York University, New York City, NY USA

**Keywords:** Intervention adaptation, Theater testing, Latina adolescent, Adolescent sexual and reproductive health, Community-based participatory research

## Abstract

**Background:**

Floreciendo is a sexual and reproductive health program for Latina teens (14–18 years) and their female caregivers adapted from the evidence-based IMARA intervention. We report on our experience theater testing Floreciendo during the preparation phase of the multiphase optimization strategy (MOST) framework. Floreciendo includes four two-hour sessions (i.e., intervention components). Our aims were to: (1) examine the preliminary acceptability, appropriateness, and feasibility of the intervention components, including the acceptability of the implementation plan (i.e., logistics, strategies), and (2) systematically report on curriculum modifications made based on findings.

**Methods:**

Using a community-based participatory research approach, we theater tested the program at a community organization over one weekend with three teen-caregiver dyads (*n* = 6) using mixed methods. Immediately following the delivery of each intervention component, teens and caregivers completed surveys and engaged in feedback sessions. Observers (*n* = 8) and facilitators (*n* = 2) completed surveys, recorded activity start and end times, and participated in a post-program discussion. Survey item ratings were on four-point Likert scales, with higher scores indicating more favorable results. Feedback informed subsequent curriculum modifications, which were documented using the FRAME.

**Results:**

We found high satisfaction with the intervention components among all surveyed (*n* = 16) and with the implementation plan among teens and caregivers (*n* = 6) (≥ 3.7/4.0). Teens and caregivers described sessions as “educational,” “motivating,” “interactive,” and “fun”; all (100%; *n* = 6) reported that they would recommend the program to others. Teens and caregivers rated the appropriateness of the material and language/wording highly (4.0/4.0; *n* = 6), although caregivers expressed difficulty understanding “passive communication” given translation difficulties. Feasibility was also rated highly across groups (≥ 3.8/4.0; *n* = 16); 18% of activities were 10 + minutes longer than planned based on observer reports but the sessions overall remained within 2 min of the allotted time. We modified the intervention components based on the feedback received. For example, we moved discussions about sex to come later in the foundational session to increase participant comfort.

**Conclusions:**

Findings offer preliminary evidence of Floreciendo’s acceptability, appropriateness, and feasibility. Theater testing is a valuable tool for intervention adaptation and FRAME is useful for tracking curriculum modifications over time. MOST researchers could consider theater testing while carrying out preparation-phase activities.

**Supplementary Information:**

The online version contains supplementary material available at 10.1186/s12889-025-22233-1.

## Background

Despite a long history of adapting interventions for new populations, careful attention to the adaptation process is only recently gaining attention in the field of implementation science [1, 2]. The term “adaptation” has numerous definitions [3]. We define adaptation as describing modifications to an intervention’s content and/or implementation strategies (i.e., the actions or processes used to deliver the intervention [4]) to enhance their fit within a given context [5]. One tool for intervention adaptation is a pretesting method called *theater testing* [6]. The goal of theater testing is to generate feedback on the material, content, and delivery of the intervention—and identify materials and/or activities missing that should be added—to enhance the intervention’s relevance, fit, and effectiveness in the new population or setting [6]. Often used to test products (e.g., advertisements, videos), theater testing has increasingly become common in the social sciences sphere since it was proposed as a centerpiece in the widely employed ADAPT-ITT model for intervention adaptation [6, 7].

Theater testing involves demonstrating an intervention to participants and requesting their immediate reactions, with key contributors and program staff observing and also contributing their ideas on potential adaptations [6]. Given its ability to solicit real-time feedback, from an implementation science perspective, theater testing may serve as a valuable tool to provide a preliminary assessment of an intervention’s acceptability, appropriateness, and feasibility. These three implementation outcomes (i.e., acceptability, appropriateness, and feasibility) are considered “leading indicators” of implementation success [8]. Examining these outcomes before full-scale implementation allows for intervention refinement that can enhance adoption (i.e., uptake [9]) and sustainability (i.e., maintenance/institutionalization [9]).

Despite growing interest in adaptation among implementation scientists and the popularity of the ADAPTT-ITT model [7], literature describing adaptation processes—and theater testing specifically—is still developing. Adaptation processes are also rarely documented among studies using the multiphase optimization strategy (MOST) framework [10], a translational framework for the development of interventions [11]. The MOST framework supports the creation of an intervention that is both maximally effective and implementable (e.g., affordable, scalable, and efficient). The framework involves separating out parts of the intervention (i.e., intervention components) for testing prior to conducting an evaluation randomized controlled trial (RCT) of the “bundled” intervention. Three phases comprise the framework: (1) identifying and piloting candidate intervention components (preparation phase); (2) experimentally testing the components to inform decisions about which to include in the optimized intervention (optimization phase); and (3) rigorously assessing the optimized intervention (evaluation phase) [11].

This paper helps to fill key gaps in the literature by describing our experience theater testing Floreciendo—a sexual and reproductive health program for Latina teens and their female caregivers (i.e., mothers, sisters, aunts, grandmothers)—during the preparation phase of the MOST framework. Over two years and using a systematic process, our team adapted Floreciendo from IMARA (Informed, Motivated, Aware, and Responsible about AIDS), an evidence-based intervention for Black 14–18 year-old teen girls and their mothers [12, 13]. We began the adaptation process by conducting formative research to inform the content and structure of Floreciendo. Based on our findings, we adapted IMARA’s two-day format to include four two-hour sessions, serving as our intervention components in the MOST framework. We also adapted the intervention content for Floreciendo, for instance, by adding content on unplanned pregnancy and on the influence of gender norms and family expectations in Latine culture on teens’ sexual and reproductive health practices. Adaptations made during this initial round of data collection are reported in detail elsewhere [14]. Following this initial round of data collection, we conducted additional formative research to understand implementation considerations (e.g., when the program should be delivered, desired characteristics of facilitators, etc.), which informed our creation of an implementation plan for Floreciendo [15].

Once we had generated a full draft of the curriculum and an implementation plan, we wished to theater test Floreciendo, which is the focus of the current paper. Our immediate goal was to further refine the content of the four curricular sessions (i.e., the intervention components) and the implementation plan prior to carrying out a pilot optimization trial of Floreciendo across community organizations in the Chicagoland area. Our study aims were to: (1) assess the preliminary acceptability, appropriateness, and feasibility of the intervention components, including the acceptability of delivery logistics/implementation strategies, and (2) document curriculum modifications made using a systematic reporting tool, informed by our findings. Our long-term goal is to arrive at an effective and implementable intervention that achieves adoption and sustainability within community organizations.

## Methods

### Study design and outcomes of interest

We used a convergent parallel mixed methods design, meaning that quantitative and qualitative data were simultaneously collected and analyzed separately with equal weight given to both (notated as QUAN + QUAL) [16]. We triangulated findings across methods to achieve the study aims [16]. In line with Proctor’s implementation outcomes framework [9], we were interested in: (1) *acceptability*: the perception that Floreciendo’s content and implementation plan are agreeable, palatable, or satisfactory; (2) *appropriateness*: the perceived fit, relevance, or compatibility of Floreciendo among Latina teens and female caregivers; and (3) *feasibility*: the extent to which Floreciendo can be successfully implemented within a community organization setting.

### Study team and positionality statement

We used a community-based participatory research (CBPR) approach [17] in this study. The Kedzie Center, Centro Romero, the Center for Dissemination and Implementation Science at the University of Illinois Chicago, and the Floreciendo Community Advisory Council partnered to support Latina teens with their sexual and reproductive health (see Merrill at al. for details; [14, 15]). Our 20-person authorship team for this study is comprised of 9 university researchers, 7 community organization representatives, 2 experts in curriculum development, 1 Latina teen, and 1 female caregiver of a Latina teen. Most of our team members (90%) identify as female and 13 (65%) as Latina. Twelve co-authors are members of Floreciendo’s Community Advisory Council. Together, we have expertise in Latina health, the Latine experience, sexual and reproductive health, family-based interventions, curriculum development, intervention adaptation, implementation science, and the MOST framework (note that we use *Latina* to refer to people who identify as female and *Latine* to refer to people of varying genders, both of Latin American heritage). We acknowledge that our positionalities have shaped how we each approached this study. Our numerous meetings and correspondences facilitated extensive discussion about the framing of our findings, thereby strengthening its rigor [18].

### Intervention overview

The Floreciendo program aims to reduce risky sexual behavior, incidence of STIs, and unplanned pregnancies among Latina teens. Its four 2-hour workshop sessions (eight hours in total) focus on the following key objectives:

- *Foundations in sexual risk prevention*: Build a foundation of knowledge in sexual and reproductive health (e.g., in STIs, condoms, contraception) and develop an awareness of sexual behavior risks;
- *Condoms and contraception*: Learn about different forms of condoms and contraception and how to use them;
- *Family strengthening*: Understand how to communicate with their teen/caregiver about sex; discuss family norms and expectations in Latine culture;
- *Gender and relationships*: Learn about what a healthy relationship looks like and how to recognize intimate partner violence (IPV); discuss how gender and cultural norms in Latine culture influence teens’ sexual behavior.

Teens and caregivers start each session together for introductory activities, then meet in separate groups while covering parallel content, and return to the large group to participate in joint activities. This session structure is designed to give teens and caregivers separate spaces to learn without judgment before interacting with each other. It also seeks to enhance caregivers’ credibility as a resource for sexual risk behavior. The session structure aligns with that used by IMARA [12, 13] and with feedback obtained during our adaptation process for Floreciendo [14].

Based on feedback from Latina teens, female caregivers, and community organization staff in qualitative data collected [15], our team developed an implementation plan for intervention delivery as follows:

- *Timing*: We hosted the workshop on weekend days (Saturday to Sunday).
- *Facilitators*: We identified two Latina facilitators to deliver the workshop—one to work mostly with teens and the other with caregivers. For theater testing, we trained facilitators from the university team with the expectation that we would train facilitators from community organizations in future phases of the study.
- *Language*: We ensured that facilitators would be prepared to deliver workshop sessions in English and/or Spanish, depending on participants’ preference.
- *Setting/space*: We ensured that two quiet and private rooms would be available for separate and joint sessions.
- *Handouts and posters*: All participants received handouts specific to each workshop session, including a list of local resources (e.g., sexual and reproductive health services available). Posters were printed to support delivery of the session content.
- *Food*: We provided breakfast and lunch on each workshop day.
- *Sexual risk prevention kits*: We provided small cosmetic bags to teens with sexual and reproductive health products (e.g., condoms, lube, tampons).
- *Graduation*: At the end of the four sessions, a small graduation ceremony was held. Each participant received individual praise and a certificate of completion.
- *Childcare*: We provided childcare for caregivers’ younger children.
- *Stipend and transport reimbursement*: Participants were given $200 cash as reimbursement for their time and transport to and from the site.

### Participants and procedures

To be eligible to participate in theater testing, teens had to identify as Latina and be 14–18 years old. Caregivers had to be the mother or female caregiver (e.g., aunt, sister, grandmother) of a Latina teen (14–18 years old), be 19 years or older, and be invited by an eligible teen. Both teens and caregivers had to speak English and/or Spanish, be available for the workshop, and be living with or in daily contact with each other.

We hosted the theater testing in July 2023 at one of our community partner sites over a weekend (i.e., sequential Saturday and Sunday days). Two sessions were delivered on Saturday and two on Sunday. During separate sessions (i.e., when teens and caregivers are split), the teen facilitator used English, and the caregiver facilitator used Spanish, based on participants’ preference. During joint sessions (i.e., when teens and caregivers are together), facilitators began by using simultaneous translation but then switched to Spanish based on participants’ preference. Participants completed brief feedback surveys and engaged in moderated discussions immediately following each session. Eight observers from the research team and community partner organizations completed surveys while sitting in on each session. Facilitators completed surveys similar to those of observers at the end of each workshop day. Following the workshop, observers and facilitators provided additional thoughts during a feedback discussion.

### Quantitative and qualitative tools

We measured the implementation outcomes on surveys administered after each session as follows, adapting survey items from previous studies of IMARA for context [12, 19]. All items were rated on a four-point Likert scale from strongly disagree to strongly agree:

- *Acceptability (all surveys)*: On teen and caregiver surveys, we measured the extent to which they liked each activity (number of items varied by session). We also included five items to measure how much they liked participating in the session as a whole, found the information useful and easy to understand, found the session to be fun, and felt comfortable participating in the session. On observer and facilitator surveys, we measured the extent to which participants were engaged in each activity (number of items varied by session). We also included three items to measure participant engagement in the session as a whole, participant comfort participating in the session, and perceived comfort with the facilitators.
- *Feasibility (all surveys)*: On all surveys, we measured perceptions of whether the session had run smoothly (one item). One observer in each group (teens and caregivers) also recorded the start and end times of each activity.
- *Appropriateness (teen and caregiver surveys only)*: On teen and caregiver surveys, we measured the extent to which they perceived the material and the language/wording to be appropriate for Latina teens (14–18 years old) (two items).

Surveys offered an opportunity to provide additional feedback on content missing from the session or content that should be removed, challenges with the content’s delivery, and suggestions for improving the session. At the conclusion of the program, teens and caregivers reported their satisfaction with the implementation plan (12 items on a four-point Likert scale from very satisfied to very unsatisfied) (see *Floreciendo Overview* above) and participated in feedback sessions for expanded discussion on the topics addressed in the surveys. Feedback sessions were audio recorded, transcribed, and translated where needed.

### Data analyses

Descriptive statistics were used to summarize the quantitative survey data. Two team members (JA and IH) coded the qualitative data from the open-ended survey questions and feedback session transcripts using a rapid analysis approach, which was checked for completeness by the first author (KM) [20, 21]. Findings from qualitative data were directly compared with the quantitative results to facilitate the merging of findings [22]. Over several meetings, preliminary results were discussed in detail and refined with our wider CBPR team, which included the observers and facilitators and thus enabled member checking— i.e., whereby participants in the research process check the results for their accuracy and reliability [23]. We observed few differences in acceptability, appropriateness, or feasibility across the four curriculum sessions and thus report consolidated findings across sessions.

We made curriculum modifications based on our findings. These curriculum modifications built on an earlier round of curriculum modifications made when adapting the IMARA program for Latinas (publication forthcoming). We documented the modifications using the Framework for Reporting Adaptations and Modifications- Enhanced (FRAME) [5]. The FRAME provides a structured approach to reporting when modifications occur, whether or not modifications are planned, who participated in the decision to modify, the goal of the modification, what is modified, at what level of delivery the modification is made, what is the nature of the modification, and the relationship of the modification to fidelity [5]. Two team members (KM and JS) coded the modifications according to the FRAME and generated a detailed report on the specifics [5].

### Ethical considerations

We obtained assent and parent permission from minors (i.e., under 18 years) and consent from adult teens and female caregivers. This research was approved by the institutional review board at the University of Illinois Chicago (IRB 00000116).

## Results

### Participant demographics

Three teen-caregiver dyads participated in theater testing. Teens ranged in age from 14 to 16 years (mean = 15 years) and spanned across 9-11th grade. They self-identified as being of Mexican (67%) and Guatemalan (33%) descent and mostly of first-generation immigration status (67%). Caregivers self-identified as biological mothers (67%) and adoptive mothers (33%) of their teens, of Mexican, Guatemalan, and Puerto Rican descent, and mostly of first-generation immigration status (67%). Caregivers ranged in education level from having some high school schooling with no diploma to having a Masters’ degree. All participants reported primarily speaking Spanish at home and having an annual household income of less than $50,000.

### Acceptability

Session acceptability ratings were high across groups (i.e., teens, caregivers, observers, and facilitators) and sessions (≥ 3.7/4.0) (*n* = 16) (Table 1). Teens and caregivers liked the curriculum content and found the material useful. They indicated that much of the Floreciendo content was new to them. For example, teens noted that they had never been taught how to use condoms in their sex-ed courses at school. On surveys, teens and caregivers described the activities as *“educational*,*” “motivating*,*” “engaging*,*” “interactive*,*”* and *“fun.”* Overall, teens, caregivers, observers, and facilitators liked the combination of interactive games (e.g., True/False), demonstration and practice activities (e.g., putting external condoms on penis models), role plays (e.g., between teens and caregivers), and group discussions (e.g., about healthy and unhealthy relationships). Some topics prompted deep conversations, which allowed teens and caregivers to be *“vulnerable”* and *“emotional”* and *“listen to each other in a new way”* (observer surveys).

Proposed revisions to the curriculum content were provided (see *Curriculum Modifications* below). For example, observers and facilitators recommended that discussions about the reasons people choose to have sex be moved later in the session when participants would feel more comfortable sharing their views. Likewise, caregivers requested additional content and emphasis on IPV, especially psychological violence. Caregivers and observers recommended activities addressing gender norms in Latine culture discuss ways to change the norms.

Workshop logistics received high satisfaction ratings among teens and caregivers (scores averaged at least 3.7/4.0) (*n* = 6) (Table 2). Hosting the workshop on a single weekend was generally acceptable, although some felt it was too long and recommended spacing sessions over two weekends. Teens and caregivers liked the location, stipend, and small group size, which felt intimate. Workshop facilitators received high praise for how they engaged participants and created a comfortable environment. Teens liked that the facilitators trusted them to be able to have conversations about sensitive topics. Teens and caregivers enjoyed the inclusion of individual praise when distributing certificates during the graduation ceremony, and teens appreciated receiving the sexual risk prevention kits. Constructive feedback was given on the posters and handouts (e.g., posters were cumbersome, handouts needed page numbers) and on the food provided during the workshop (e.g., consider different diets).

All teens and caregivers (100%; *n* = 6) reported that they would recommend the program to others. In open-ended survey responses, they wrote:

*You are able to learn and experience how to use different methods to have safe sex*,* such as how to put on condoms (external and internal) and how to react to things peacefully and calmly. Overall*,* I think this was a good educational program.* (Teen)

*The information that was shared is very important and useful to inform the teenagers and also us as parents.* (Caregiver)

### Appropriateness

Ratings of the appropriateness of the material and language/wording averaged 4.0/4.0 among teens and caregivers (*n* = 6) (Table 1). Teens and caregivers indicated that the program was well-suited to Latine culture and generally agreed that activities that prompt discomfort should be included in the curriculum. For example, whereas one teen described the condom demonstrations as *“weird*,*”* another teen explained, *“Discomfort is part of the process when learning about sex. The whole point is stepping out of your comfort zone.”* One caregiver expressed concerns with educating teens about gender identity, saying, *“Kids are confused these days because of all the pronouns and are too young to be identifying themselves. They are adolescents and can change their minds.”* However, the other caregivers and teens found the activity to be educational and important to include.

Regarding the appropriateness of the language and wording used in the curriculum, caregivers struggled to understand the difference between passive and assertive communication due to translational challenges. *Pasiva* in Spanish can translate to “calm” or “patient,” which caregivers considered a desirable communication quality. Observers recommended using submissive or *sumisa* to distinguish it from assertive communication. In other cases, the language and wording were considered well-suited to the population.

### Feasibility

Feasibility ratings on how smoothly the sessions ran averaged 4.0/4.0 among teens and caregivers (n = 6) and 3.8/4.0 among observers and facilitators (n = 10) (Table 1). While 18% of activities ran 10 + minutes over or under time (12/64), the average delivery time of sessions remained within 2 minutes of the 2 hours allotted per session (Supplementary Material 1). In the caregiver group, two activities were skipped because discussions about mental health and teens’ sexual behavior ran long. One observer attributed the skipped activities to the difficulty of following time constraints while still allowing for discussion—particularly among caregivers who *“tend to talk more.”* Additional facilitator training was recommended to achieve this balancing act. Time management challenges were also attributed to the initial use of simultaneous translation in English and Spanish during joint sessions. After facilitators asked participants about their preferred language in joint sessions and they indicated Spanish, implementation went more smoothly. An observer recommended asking about language preference before each workshop in the future. A final challenge to how smoothly the sessions ran pertained to the use of posters, which were perceived by observers and facilitators as too large and too numerous. Despite these challenges, an observer summarized the general feeling that the sessions had run smoothly: *“Although timing may have gone over*,* the pacing rarely felt off.”*

**Table 1:** Ratings of acceptability, appropriateness, and feasibility of workshop content by teens and caregivers (*n* = 6) and observers and facilitators (*n* = 10)

	Teens	Caregivers	Representative quotes
	Mean (SD)		
**Acceptability**			
1) I liked participating in this session as a whole.	3.9 (0.3)	4.0 (0.0)	*“[The Virus Carrier Handshake activity] is a fun way to meet each other and learn how people can get an STI.”* (Teen)
2) The information was useful to me.	4.0 (0.0)	4.0 (0.0)	
3) The information was easy to understand.	3.9 (0.2)	3.8 (0.4)	
4) The session was fun.	3.9 (0.2)	4.0 (0.0)	*“[The parental monitoring activity] makes us feel more confident when we monitor this topic with our daughters.”* (Caregiver)
5) I felt comfortable participating in this session.	3.9 (0.3)	4.0 (0.0)	
**Appropriateness**			
1) The material is appropriate for Latina teens (14–18 years old).	4.0 (0.0)	4.0 (0.0)	*“Weird.”* (Teen)
2) The language/wording is appropriate for Latina teens (14–18 years old).	4.0 (0.0)	4.0 (0.0)	*“Discomfort is part of the process when learning about sex. The whole point is stepping out of your comfort zone.”* (Teen)
**Feasibility**			
1) The session ran smoothly.	4.0 (0.0)	4.0 (0.0)	None.

	Observers & facilitators		Representative quotes
	Mean (SD)		
**Acceptability**			
1) Participants were engaged in this session as a whole.	3.8 (0.3)		*“[In the Condoms & Contraception session] it was great that they had the chance to touch and practice with their own hands.”* (Observer)
2) Participants were comfortable participating in this session.	3.7 (0.4)		
3) Participants were comfortable with the facilitators in this session.	3.8 (0.3)		*“Teens were honest. Caregivers were vulnerable.”* (Observer)
**Feasibility**			
1) The session ran smoothly.	3.8 (0.2)		*“Prepare facilitators for how to address off-topic personal questions.”* (Observer)

Notes: SD = standard deviation. Ratings are averaged across the four sessions on a four-point scale: 1 = Strongly disagree; 2 = Somewhat disagree; 3 = Somewhat agree; 4 = Strongly agree

**Table 2:** Satisfaction ratings with workshop logistics and implementation strategies as reported by Latina teens (*n* = 3) and female caregivers (*n* = 3), with representative quotes

Logistics and strategies	Teens	Caregivers	Representative quotes
Hosting the workshop on a full weekend (Saturday and Sunday)	3.7 (0.6)	4.0 (0.0)	Days were “*long*” and *“like school”* (Teen) but the material felt *“fresh”* and there was good *“momentum”* (Observer)
The workshop facilitators	4.0 (0.0)	4.0 (0.0)	*“Super nice and friendly”* (Teen); *“I was impressed”* (Caregiver); *“Although young*,* very mature and easy to talk to”* (Caregiver)
The rooms where the sessions took place	4.0 (0.0)	3.7 (0.6)	*“A nice*,* comfortable*,* quiet space”* (Teen)
The handouts and posters	3.7 (0.6)	4.0 (0.0)	A *“useful source all in one place”* (Teen). *“Staple all handouts and give them page numbers”* (Observer)
Workshop food (breakfast, lunch)	3.7 (0.6)	3.7 (0.6)	*“Very tasty”* (Caregiver); *“More consideration of different diets”* (Observer)
The sexual risk prevention kits (for teens)	4.0 (0.0)	4.0 (0.0)	*“Cute color”* and *“We can bring it to school”* (Teen)
The graduation, including the certificates	4.0 (0.0)	4.0 (0.0)	*“Sincere and a good way to wrap up the curriculum”* (Observer)
The stipend (for participation and transport)	4.0 (0.0)	3.7 (0.6)	*“Wonderful*,* although I am doing this to further educate myself*,* not for the money”* (Caregiver)

Notes: Ratings are on a four-point scale: 1 = Very unsatisfied; 2 = Somewhat unsatisfied; 3 = Somewhat satisfied; 4 = Very satisfied. Caregivers were not asked about satisfaction with the childcare provided

### Curriculum modifications

Based on these findings, we made 16 modifications to the curriculum (Table 3). We added content on mental health, developmental changes during puberty, contraception side effects, and forms of IPV. We revised the ordering of some activities. For example, we moved discussions about sex to come later in the Foundations in Sexual Risk Prevention session and integrated content on IPV and consent to more directly build on the content about healthy and unhealthy relationships. We addressed the translation concerns about passive (“pasiva”) communication styles by changing the terminology to effective and ineffective communication styles. We provided handouts with a list of health and social resources across the Chicagoland area, tampon use and care, and PreEP to meet the need for access to care and address topics that are not covered extensively throughout the curriculum. We acquired fidget toys for teens to help them feel more comfortable during workshop sessions. We shortened the number of posters needed during the workshop sessions by instead providing the information in handouts printed for teens and caregivers.

**Table 3:** Curriculum modifications made based on feedback received and reported using FRAME

Session	Nature of modification	Description of modification	Who suggested the adaptation?	Representative quotes
Foundations in Sexual Risk Prevention	Adding elements; Tailoring/ rewording/ refining	• Added content on influences on our mental health • Removed Young and Latina activity and integrated content into other areas • Added content on developmental changes during puberty for teens • Added an example of a personal story for the facilitator to share with teens • Updated the parental monitoring example to focus on social media • Added a handout on how to use tampons and on PrEP	Teens, Observers	*“The workshop should also teach how to use a tampon”* (Teen) *“Motivate [teens] to interact with each other and communicate”* (Teen) *“The personal story was entertaining and allowed the teens to connect more with others (i.e. the facilitators/each other)”* (Teen) *“Make [parental monitoring] more engaging”* (Observer)
Condoms & Contraception	Adding elements; rewording/ refining	• Clarified definitions for key terms (e.g., “external condom,” “internal condom”) • Added a note about contraception side effects	Observer	*“Creating space for [side effects associated with birth control use] in the curriculum would be helpful”* (Observer)
Gender and Relationships	Adding elements; rewording/ refining	• Added definition for identity • Clarified healthy/unhealthy relationship scenarios • Added an activity (the power and control wheel) to explain IPV • Added definitions for the forms of IPV, with a focus on psychological violence	Teens, Caregivers, Observers	*“Be a bit more clear on what identities you are talking about.”* (Teen) *“Better examples/scenarios in this section needed”* (Observer) *“Indicate what is the cycle of control and power of abuses”* (Caregiver)
Family Strengthening	Adding elements; rewording/ refining	• Added an activity for teens and caregivers to give each other praise • Changed wording from *passive*,* aggressive*,* and assertive* to *effective and ineffective* communication styles • Added role plays focused on birth control and IPV • Provide fidget toys to participants during an activity focused on teen/caregiver sharing	Observers	*“Highlight…what makes something assertive communication versus passive communication”* (Observer) *“Change translation of ‘passive’”* (Observer) *“Teens’ behavior changed when they were listening to the caregivers: they seemed nervous”* (Observer)

Notes: Other FRAME reporting categories apply to all modifications in the table as follows: (1) WHEN did the modification occur? Post-implementation during theater testing; (2) Were adaptations PLANNED? Proactive; (3) What was the GOAL? To address cultural factors and improve the appropriateness or fit of the intervention for Latinas; (4) At what LEVEL OF DELIVERY (for whom/what is the modification made)? Intervention participants; (5) Relationship to FIDELITY or core elements? Fidelity preserved

## Discussion

This study reports on our experience theater testing Floreciendo, a four-session intervention for Latina teens and their female caregivers. We found support for the acceptability, appropriateness, and feasibility of the intervention components across Latina teens, their female caregivers, observers, and facilitators. Latina teens and their female caregivers liked the sessions and delivery logistics and would recommend Floreciendo to others. The program was viewed as appropriate and feasible to implement within a community organization setting. These positive findings bode well for the future delivery of the program [8].

Our study demonstrates the value of theater testing as a tool to adapt a sexual and reproductive health program for youth, supporting the limited but growing literature in this area [24–26]. Theater testing facilitated real-time feedback from Latina teens and their female caregivers, which is a notable strength of the method. Engaging the target population in the adaptation process is critical to improve program fit [27, 28]. Observers, facilitators, and community representatives also added depth to the feedback from teens and caregivers in line with our CBPR approach [17]. Community organizations are a key provider of STI/HIV prevention services to minoritized populations [29, 30] and their perspectives are thus essential to long-term sustainability and scale-up [25] of sexual and reproductive health programming.

Researchers using the MOST framework could consider theater testing as a method to support their preparation-phase activities. The *preparation* phase of MOST, although providing the foundation for the *optimization* and *evaluation* phases, has to date received less attention than the other phases of the framework [31]. While researchers have begun to explore tools that can strengthen intervention component development during the preparation phase of MOST (e.g., human centered design [32]), theater testing is an untapped tool for this purpose. Theater testing provided an inclusive and efficient manner of refining the intervention components, which we are currently testing in a pilot optimization trial of Floreciendo.

Our work further adds to the growing literature on documentation of program adaptations. Feedback obtained and challenges encountered during theater testing (e.g., translation issues with the word *pasiva*, etc.) informed our curriculum modifications, which we documented using the FRAME [5]. Our reporting of curriculum adaptations in this paper using the FRAME builds on our extensive documentation of program adaptations prior to implementation [14]. In doing so, our work strengthens the limited literature reporting on adaptations over time, which is necessary for developing a fuller understanding of how interventions change and facilitating future exploration of the influence of such adaptations on desired outcomes [1, 33].

Study limitations must be noted. Three Latina teens-female caregiver dyads participated in the theater testing, which is a small sample size but aligns with our planned size for Floreciendo groups. Further, feedback from eight observers, two facilitators, and the Community Advisory Council ensured that a wide range of perspectives informed curriculum decisions made following the theater testing. Teen participants were on the low end of the 14–18 year age range (i.e., aged 14–16 years) and hence we did not get feedback from teens ages 17–18 years. Still, we do not anticipate the feedback to be notably different by age. Our quantitative measures of acceptability, appropriateness, and feasibility were not drawn from psychometrically validated measures, but they were adapted from measures used in previous studies of IMARA [12, 19].

## Conclusions

This study demonstrates the value of theater testing as a method for strengthening intervention adaptation and the preliminary assessment of implementation outcomes, including within studies using the MOST framework. We found early support for the acceptability, appropriateness, and feasibility of the Floreciendo intervention components and documented our post-implementation curriculum modifications using the FRAME. Findings from this study build on our previous work to systematically adapt IMARA for a Latina population [14, 15] and informed a pilot optimization trial of Floreciendo using the MOST framework that is currently underway.

## Electronic supplementary material

Below is the link to the electronic supplementary material.

Supplementary Material 1

## Data Availability

Data are available on reasonable request from the corresponding author (KM).

## Abbreviations

MOST framework: Multiphase optimization strategy framework
FRAME: Framework for Reporting Adaptations and Modifications- Enhanced
CBPR: Community-based participatory research
RCT: Randomized controlled trial
IMARA: Informed, Motivated, Aware, and Responsible about AIDS
IPV: Intimate partner violence
STI: Sexually transmitted infection
