# Supplementary material for: Theater testing a sexual and reproductive health program for Latina teens and their female caregivers: a mixed methods study
Source: BMC Public Health. 2025 Jul 7;25:2399. doi: 10.1186/s12889-025-22233-1 (PMC12232844; doi:10.1186/s12889-025-22233-1)
Supplement: Supplementary file 1 — Supplementary Material 1 [file 12889_2025_22233_MOESM1_ESM.docx]

**Theater testing a sexual and reproductive health program for Latina teens and their female caregivers: A mixed methods study**

**Supplementary Table 1**: Tracking actual versus planned delivery timing of session activities

|  | **Teens** | | | **Caregivers** | | |
| --- | --- | --- | --- | --- | --- | --- |
|  | **Planned duration** | **Actual duration** | **Difference** | **Planned duration** | **Actual duration** | **Difference** |
| **Foundations in Sexual Risk Prevention** | | | | | | |
| Program Introduction and Contract | 15 | 19 | 4 | 15 | 12 | -3 |
| Virus Carrier Handshake | 10 | 13 | 3 | 10 | 14 | 4 |
| Mental Health and Feeling Thermometer Check-in | 10 | 18 | 8 | 10 | 18 | 8 |
| Young and Latina | 15 | 17 | 2 | 15 | 32 | 17 |
| High, Low, or No Risk | 15 | 14 | -1 | 15 | Not delivered | N/A |
| True or False Game | 20 | 8 | -12 | 20 | Not delivered | N/A |
| Personalizing Risky Situations/Parental Monitoring | 20 | 25 | 5 | 20 | 26 | 6 |
| Pair & Share | 10 | 13 | 3 | 10 | 8 | -2 |
| Key Messages | 5 | Missing | N/A | 5 | 2 | -3 |
| *Mean difference* |  |  | 2 |  |  | 4 |
| **Condoms and Contraception** | | | | | | |
| Intro and feeling thermometer check-in | 10 | 3 | -7 | 10 | 2 | -8 |
| External Condoms (LIPSTICK) (teens & caregivers separate) | 20 | 19 | -1 | 20 | 20 | 0 |
| Internal Condoms | 15 | 13 | -4 | 15 | 10 | -5 |
| Dental Dams | 10 | 5 | -5 | 10 | 6 | -4 |
| Dos and Don’ts of Condom and Dental Dam Use | 5 | 3 | -2 | 5 | 3 | -2 |
| Contraception | 20 | 17 | -3 | 20 | 22 | 2 |
| LIPSTICK (teens & caregivers together) | 25 | 11 | -14 | 25 | 11 | -14 |
| Pair & Share | 10 | 4 | -5 | 10 | 4 | -6 |
| Key Messages | 5 | 3 | -2 | 5 | 2 | -3 |
| *Mean difference* |  |  | -5 |  |  | -4 |
| **Family Strengthening** | | | | | | |
| Intro and Feeling Thermometer Check-in | 10 | 3 | -7 | 10 | 2 | -8 |
| Passive, Aggressive, Assertive Communication | 15 | 16 | 1 | 15 | 26 | 11 |
| Separate Assertive Communication | 15 | 16 | 1 | 15 | 11 | -4 |
| Joint Assertive Communication Role-Play | 30 | 20 | -10 | 30 | 20 | -10 |
| Family Norms and Expectations | 30 | 56 | 26 | 30 | 45 | 15 |
| Pair and Share | 15 | 5 | -10 | 15 | 4 | -11 |
| Key Messages | 5 | 8 | 3 | 5 | 1 | -4 |
| *Mean difference* |  |  | 1 |  |  | -1 |
| **Gender & Relationships** | | | | | | |
| Intro and Feeling Thermometer Check-in | 10 | 6 | -4 | 10 | 6 | -4 |
| Our Identities (including gender identity) | 10 | 8 | -2 | 10 | 14 | 4 |
| Healthy and Unhealthy Relationships | 30 | 45 | 15 | 30 | 36 | 6 |
| Partner Communication (Keep It Simple, Sister: KISS) | 25 | 32 | 7 | 25 | 20 | -5 |
| Consent and partner violence | 15 | Missing | N/A | 15 | 16 | 1 |
| Gender Roles and Expectations in Latine Culture, and El Toxico activity | 20 | 28 | 8 | 20 | 27 | 7 |
| Pair & Share | 10 | 3 | -7 | 10 | 4 | -6 |
| Key Messages | 5 | 2 | -3 | 5 | 2 | -3 |
| *Mean difference* |  |  | 2 |  |  | 0 |

*Note*: Cells are highlighted if the difference between actual and planned delivery times was 10 minutes or greater.
